# Bridging theory-practice gaps in sedation for existential suffering: Clinical implications

**DOI:** 10.1017/S147895152610193X

**Published:** 2026-02-25

**Authors:** Teresa Moura

**Affiliations:** Faculdade de Ciências da Saúde e Enfermagem, Universidade Catolica Portuguesa, Portugal

Dear Editor,

As a specialist nurse working in an acute palliative care unit, I read with great interest the article by Correa-Morales et al. (2026) on palliative sedation for existential suffering. In this editorial, the authors highlight two critical theory-practice gaps that have profound clinical implications, particularly for bedside decision-making where premature refractoriness assessments may overlook viable treatment trajectories and affect ethical symptom management at the end of life.

The initial identified gap pertains to the premature assessment of patients, which can result in the administration of “bridge therapy” sedation for existential suffering that has not yet reached a refractory stage. Notably, this phenomenon is not addressed in the current guidelines concerning this topic. Nurses, who daily monitor symptom trajectories, often witness delayed benefits from psychosocial or pharmacological interventions, underscoring the need for refined assessment tools to guide sedation depth as well as other symptom management, as stated by Rodrigues et al. (2018).

The second theory-practice gap mentioned refers to starting sedation without trialing readily available short-onset treatments and is equally concerning. Evidence supports both structured dignity-therapy (Seiler et al. 2024) and meaning-centered psychotherapy (Rosenfeld et al. 2017) alongside rapid-acting agents like ketamine and psilocybin-assisted therapy (Campolina and De Oliveira 2025; Kim et al. 2024) as treatments for existential suffering; these complementary approaches remain underutilized despite feasibility in end-of-life settings. However, in patients nearing the end of life, with existential suffering and a short prognosis (e.g., days to weeks), multidisciplinary evaluation becomes essential. Rapid-onset interventions like psilocybin and ketamine may not provide effects quickly enough or lead to clinically significant outcomes in a timely manner, especially since their onset and integration periods often exceed the limited survival timelines of patients. In this context, interdisciplinary teams should conduct a comprehensive evaluation of the patient’s prognosis, values, and preferences, and the practicality of different therapeutic approaches (Van Der Elst et al. 2024). Regular bedside evaluations by nursing professionals are essential for reducing unnecessary burdens on patients and ensuring that comfort-focused sedation is prioritized when other treatment options are not viable.

The identified gaps pose a risk of overtreatment through sedation, which may compromise optimal symptom management and undermine patient autonomy – a fundamental principle highlighted in the revised European Association of Palliative Care (EAPC) framework on palliative sedation. This framework emphasizes the importance of a shared decision-making process that accounts for patients’ informed preferences, values, and care goals, ensuring that decisions are tailored to individual circumstances rather than adhering solely to standardized protocols (Surges et al. 2024). I commend the authors for their excellent work in this insightful article, which I greatly appreciate for its clarity and clinical relevance. Guidelines need to be urgently updated to truly bridge these gaps: literally connecting theory to bedside practice for more ethical, patient-centered end-of-life care.
